# Design and Development of a Computational Tool for a Dialyzer by Using Computational Fluid Dynamic (CFD) Model

**DOI:** 10.3390/membranes11120916

**Published:** 2021-11-24

**Authors:** Tuba Yaqoob, Muhammad Ahsan, Sarah Farrukh, Iftikhar Ahmad

**Affiliations:** School of Chemical and Materials Engineering, National University of Sciences and Technology, Islamabad 44000, Pakistan; tyaqoob.pse1@scme.nust.edu.pk (T.Y.); sarah.farrukh@scme.nust.edu.pk (S.F.); iftikhar.salarzai@scme.nust.edu.pk (I.A.)

**Keywords:** computational tool, membrane algorithm, stand-alone application, COMSOL application builder, dialysis

## Abstract

In order to reduce the hemodialysis cost and duration, an investigation of the effect of dialyzer design and process variables on the solute clearance rate is required. It is not easy to translate the in vivo transfer process with in vitro experiments, as it involves a high cost to produce various designs and membranes for the dialyzer. The primary objective of this study was the design and development of a computational tool for a dialyzer by using a computational fluid dynamic (CFD) model. Due to their complexity, only researchers with expertise in computational analysis can use dialyzer models. Therefore, COMSOL Inc. (Stockholm, Sweden) has made an application on membrane dialysis to study the impact of different design and process parameters on dialyzed liquid concentration. Still, membrane mathematical modeling is not considered in this application. This void hinders an investigation of the impact of membrane characteristics on the solute clearance rate. This study has developed a stand-alone computational tool in COMSOL Multiphysics 5.4 to fill this void. A review of the literature conducted shows that there are no suitable stand-alone computational tools for kidney dialysis. Very little work has been undertaken to validate the stand-alone computational tool. Medical staff in the hospitals require a computational tool that can be installed quickly and provide results with limited knowledge of dialysis. This work aims to construct a user-friendly computational tool to solve this problem. The development of a user-friendly stand-alone computational tool for the dialyzer is described thoroughly. This application simulates a mathematical model with the Finite Element Method using the COMSOL Multiphysics solver. The software tool is converted to a stand-alone version with the COMSOL compiler. The stand-alone computational tool provides the clearance rate of six different toxins and module packing density. Compared with the previous application, the stand-alone computational tool of membrane dialysis enables the user to investigate the impact of membrane characteristics and process parameters on the clearance rate of different solutes. The results are also inconsistent with the literature data, and the differences ranges are 0.09–6.35% and 0.22–2.63% for urea clearance rate and glucose clearance rate, respectively. Statistical analysis of the results is presented as mean with 95% confidence intervals (CIs) and *p* values 0.9472 and 0.833 of the urea and glucose clearance rates, respectively.

## 1. Introduction

A kidney is a complex bundle of semi-permeable, porous hollow fibers. When these fibers lose their ability to filter the water and toxins (ranging from small to large molecules) from the bloodstream, the patient is generally diagnosed with kidney failure [1,2,3]. Dialyzer plays the role of an artificial kidney for End-Stage Renal Failure (ESRF) patients. The hemodialysis machine is vital because the blood plasma is filtered inside the dialyzer’s hollow fibers. These hollow fibers, having a diameter of 200 nm and an active surface area of approximately 0.8–2.5 m^2^, are made of semi-permeable porous membranes [4,5,6]. The phenomena of diffusion and convection govern the transfer of solutes across the porous membrane. The clearance efficiency of porous membrane lies in dialyzer geometry, membrane characteristics, and process variables.

The mathematical analysis and enhanced computational power of computers can investigate the transport phenomena occurring inside the dialyzer while minimizing R&D’s cost. In the past 30 years, several mathematical models have been reported to translate the transport phenomena occurring in vivo. The models have been simulated using MATLAB^®^ R2020b, ANSYS Fluent^®^ 2021R1, and COMSOL Multiphysics^®^ V5. For example, Gałach et al. developed a three-compartment model to investigate the impact of peritoneal and hemodialysis on End-Stage Renal Disease (ESRD) patients. The model equations were simulated with MATLAB code ODE 45 [7,8,9]. Yamamoto et al. established that the Tortuous Capillary Pore Diffusion model (TCPDM) helps determine asymmetric membrane diffusive permeability [10,11,12]. Annan developed a two-dimensional mathematical model to study the blood and dialysate compartment’s flow characteristics across the membrane [13,14]. Islam et al. conducted a parametric study of a Polyflux 210H dialyzer using a solver based on the Finite Element Method (COMSOL Multiphysics) [15,16]. Donato et al. non-dimensionalized the hollow fiber dialyzer’s mathematical model to find factors that play a vital role in improving the dialyzer’s clearance rate [17,18]. A study was brought up by filtration of a non-Newtonian Casson fluid between two parallel permeable membranes [19]. The effect of the interplay among parameters on internal filtration is also investigated [20]. High flux hemodialyzer membranes of different average porosities were modeled. A toxin molecule’s diffusion and convection property through the membrane was observed through simulation using the Finite Element Method [21].

Due to the simulation models’ complexity, only researchers having expertise in computational analysis can use the solvers, i.e., MATLAB^®^ R2020b, ANSYS Fluent^®^ 2021R1, and COMSOL Multiphysics^®^ V5. A stand-alone application that provides precise user interphase is developed in COMSOL Application builder [22]. COMSOL Inc. has developed an application that enables studying the effect of few membrane properties and design parameters on module clearance efficiency. However, the COMSOL application has no mathematical model for the transport of solutes across the membrane, and the solutes are not defined as well. The COMSOL application cannot be used without COMSOL Multiphysics software [23]. Nevertheless, it inspires the development of a stand-alone computational tool based on a detailed mathematical membrane model.

A review of the literature conducted shows that there are no suitable stand-alone computational tools for kidney dialysis. Furthermore, very little work has been undertaken to validate the stand-alone computational tool. Medical staff in the hospitals require a computational tool that can be installed quickly and provide the results with limited knowledge of dialysis. This work aims to construct a user-friendly computational tool to solve this problem. In this study, the development of a stand-alone computational tool is described thoroughly. This topic constitutes a new domain with largely unstudied potential. It is one of the recent new areas for investigation in the field of kidney dialysis. 

Forms were created and developed further with various Form Objects to build a graphical user interphase (GUI). The actions that are not part of the default run were incorporated through command loops created under the Method node. The application toolbar was developed with the Main Window node of the Application Builder window. The computational tool enables the user to see the impact of different process variables and membrane characteristics on the clearance rate of solute (i.e., urea, glucose, endothelin, β2-macroglobulin, complement factor D, and albumin) without facing the complexity of the mathematical model. Such computational tools would have obvious advantages in a variety of areas of research in dialysis. 

## 2. Development of a Computational Tool

The governing equations and boundary conditions that describe momentum and mass transport in blood and dialysate compartments and across the membrane are published in previous research [24]. This study focused on the detailed procedure for developing a stand-alone computational tool, which is explained in the Supplementary Material of the article. 

### 2.1. Stand-Alone Computational Tool vs. COMSOL Application

**Figure 1:**
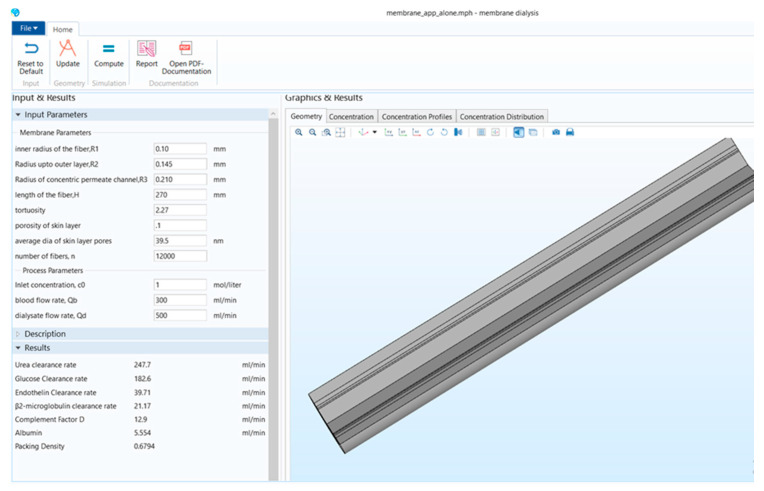
Stand-alone computational tool interphase.

**Figure 2:**
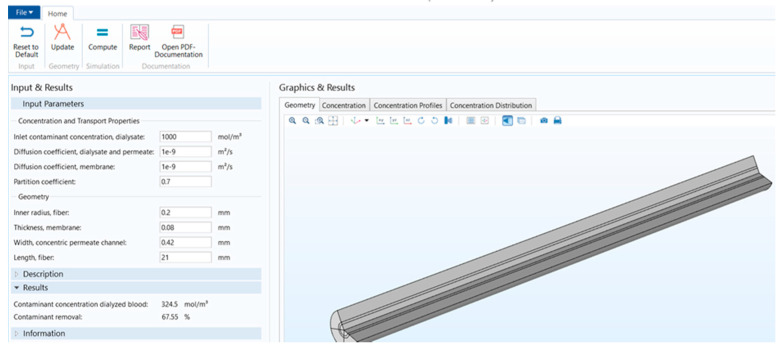
COMSOL application interphase.

Figure 1 shows that the stand-alone computational tool’s interphase has input, description, and results in a window on the left-hand side. The graphical results window is on the right-hand side, and there is a ribbon tab on the top. Figure 2 shows the user interphase of the COMSOL application, which has input parameters and results on the left-hand side and graphical results on the right-hand side. Switching from a model builder to an application builder is depicted in Figure S1.

### 2.2. Comparison of the Model Parameters

Table 1 shows the difference between the parameters used to build the mathematical models working behind these two applications.

**Figure 3:**
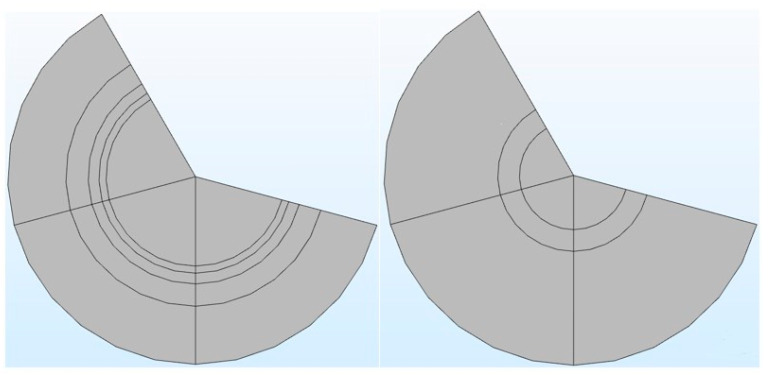
The cross-sectional view of a multi-layered membrane in a stand-alone computational tool (**left side**) vs. single-layer membrane in COMSOL application (**right side**).

It can be seen from Table 1 that in the COMSOL application, the diffusion coefficient, D(m/s), of solutes and the membrane diffusion coefficient, Dm(m/s), were taken as constant values. The diffusion coefficient of solute, D(m/s), depends on the solute’s molecular weight. The membrane diffusion coefficient, Dm, depends on the membrane’s characteristics (i.e., tortuosity τ, membrane j-th layer porosity Ɛmj, friction coefficient F(p), steric hindrance factor H_D_). Therefore, in the stand-alone application, these two parameters were mathematically modeled. The COMSOL application is built with a mathematical model, in which the average velocity on the dialysate and permeate side of the dialyzer was taken as a pre-defined numeric value. The average velocity of blood and dialysate is a function of the blood and dialysate flow rate. The blood and dialysate side velocity has been modeled with the continuity equation and the Naiver–Stokes equation in a stand-alone application. The user can change the blood and dialysate flow rate. Details of these mathematical equations are presented in a published study [24]. The experimental studies have shown that the synthetic membrane used in dialyzers are multi-layer membranes [15]. Figure 3 shows the cross-sectional view of the hollow fiber membrane in the stand-alone application and COMSOL application. That stand-alone application simulates a multi-layered (i.e., skin, middle, bulk) membrane, as evident from the cross-sectional view of the two membranes. In contrast, the COMSOL application simulates a single-layer membrane.

### 2.3. Comparison of the Input Parameters

The input parameters available in the COMSOL application and stand-alone application are shown in Table 2. It can be seen in Table 2 that there are six new parameters available in the stand-alone application, including four membrane parameters and two process parameters. These new parameters are vital in determining the clearance rate of different solutes present in the blood [24].

The comparison of results obtained from these two applications is given in Table 3. It can be seen from Table 3 that in the COMSOL application, the contaminants were not defined, and it was built by considering water on both blood and dialysate compartments. Therefore, the results obtained by the COMSOL application showed the water flux across the membrane. The dialyzer efficiency depends on the clearance rate of different solutes obtained from the dialyzer membrane [24]. Thus, the stand-alone computational tool included the clearance rate of six different solutes present in the blood. The packing density (PD) of the fibers enclosed in the dialyzer’s shell depends on the hollow fiber and annulus radius and the total number of fibers. The change in either radius (R1, R2, R3) or the number of fibers (N) not only affects the clearance rate of toxins but also impacts the packing density (PD) of the dialyzer [17]. Since PD helps to optimize the number of fibers and the dialyzer’s clearance efficiency, it is also included in the results of the stand-alone computational tool. Development of form is shown in Figures S2–S17. Table 4 presents the critical input parameters extracted from the literature for this study [10,15].

## 3. Results and Discussion

The fibers are assumed to be uniformly spaced and organized in a hexagonal order, and interstices among the adjacent annuli are neglected in the presented model. It is assumed that the viscosity of both blood and dialysate does not change with applied share. Therefore, these fluids are considered incompressible and Newtonian fluids.

**Figure 4:**
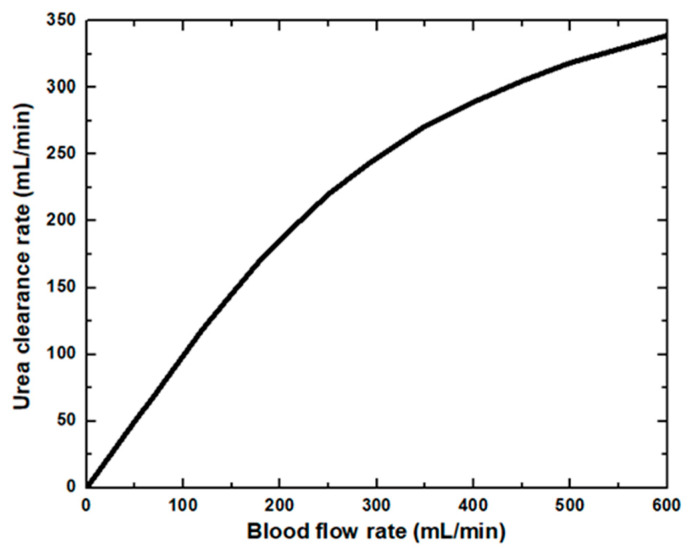
Urea clearance rate at various blood flow rates.

**Figure 5:**
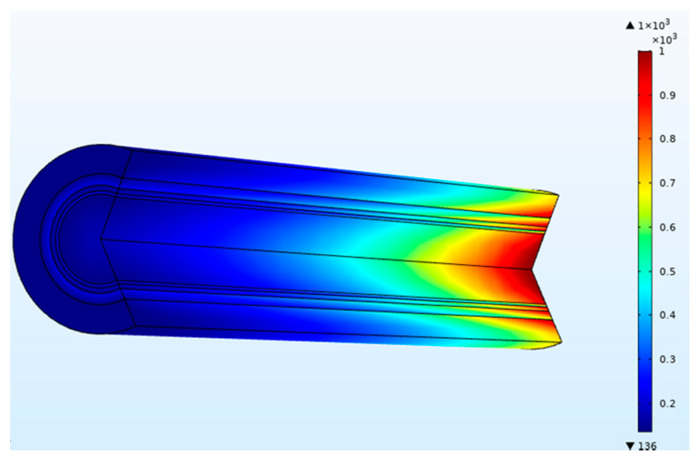
Concentration (mol/m^3^) of blood and dialysate in a three-layer membrane fiber (blood flow rate = 300 mL/min and dialysate flow rate = 500 mL/min).

In the dialyzer, the typical correlation between the blood flow rate and solute removal is curvilinear. Increasing blood flow increases solute clearance, but the increase is not proportional to the increased blood flow, as diffusion’s effectiveness decreases as blood flow rises. At low blood flow rates, the solute removal cannot surpass the blood flow rate. At higher blood flow rates, rises in clearance rates gradually reduce as the characteristics of the dialysis membrane become the limiting factor. The blood flow rate affects the clearance of small molecules and therefore is said to be flow-limited because their clearance is highly flow-dependent, as shown in Figure 4. In this study, dialysate flow rates are maintained at 500 mL/min. The urea clearance rate increases with the blood flow rate and gradually reaches a maximum value. However, at low blood flow rates, the capability of the high-efficiency dialyzer cannot be utilized, and the clearance rate is similar to that of the low-flux dialyzer. Urea and other small molecules diffuse from the blood through the pores in the membrane into the dialysate driven by an intense concentration gradient. Figure 5 demonstrates the concentration of blood and dialysate in a three-layer membrane fiber. The blood flow inside the fiber lumen is modeled with a three-dimensional finite element model. Blood is modeled as a Newtonian fluid with a dynamic inlet viscosity and density. As blood thickening due to ultrafiltration occurs along the dialyzer length, blood viscosity was assumed to increase linearly. The flow distribution in the blood and dialysate compartments of a hollow fiber dialyzer defines the mass transfer efficiency. A uniform flow distribution helps local mass transfer, and any difference caused by non-uniform flow in either the blood or dialysate compartment results in an inferior uremic solute removal from the blood. A benefit in dialyzer efficiency can be attributed to an increase in the effective membrane surface area. Fiber bundle perfusion is increased, and preferential flow channeling and fluid stagnation are impeded with higher dialysate flows. Good agreement is found when comparing results from this work against published data available in the literature [15].

**Figure 6:**
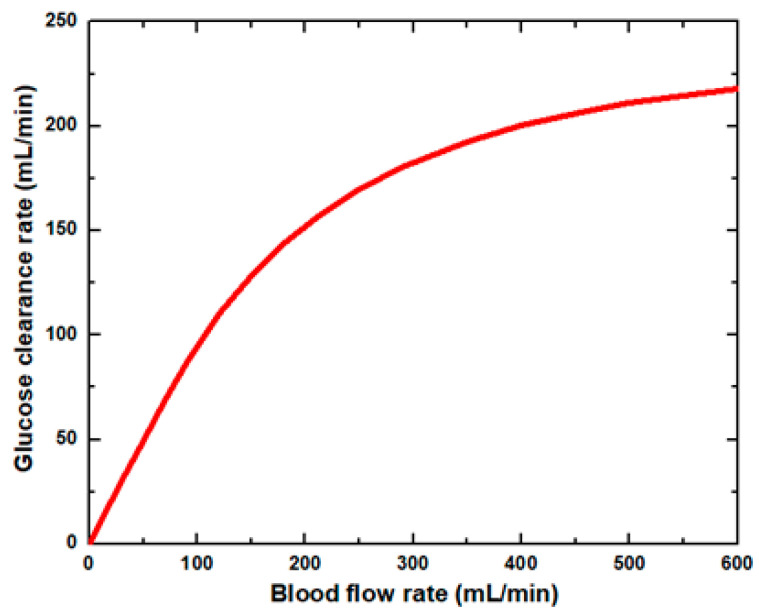
Glucose clearance rate at various blood flow rates.

**Figure 7:**
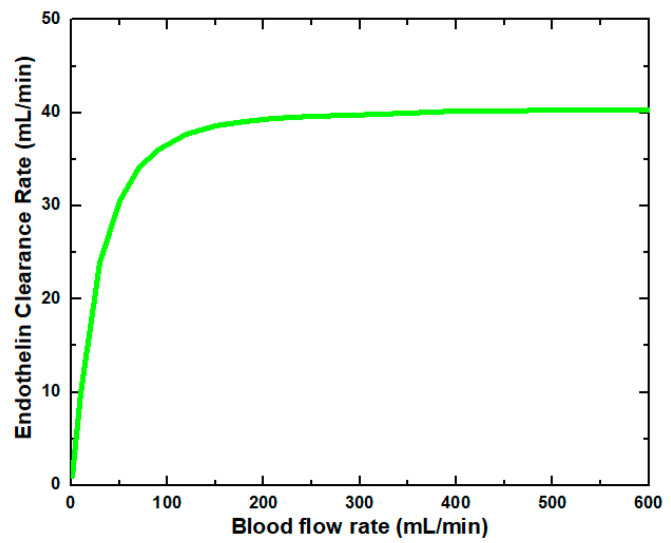
Endothelin clearance rate at various blood flow rates.

**Figure 8:**
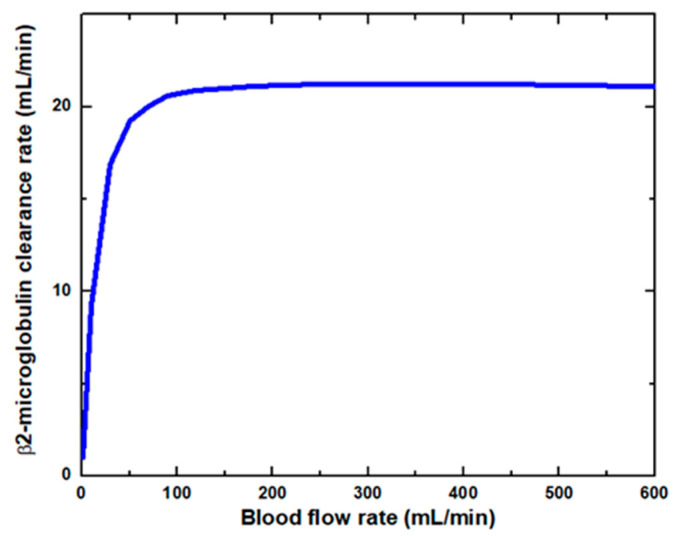
β2-microglobulin clearance rate at various blood flow rates.

Figure 6 shows that the glucose clearance rate also rises with the blood flow rate and steadily achieves the highest value. The glucose clearance rate increases fastest from a blood flow rate of 0–300 mL/min and reaches the value of 175 mL/min. The effect of blood flow rate on the glucose clearance rate decreases as the blood flow rate increases, i.e., 300–600 mL/min. Figure 7 shows that the endothelin clearance rate increases sharply and reaches 38 mL/min at low blood flow rate values, i.e., 0–100 mL/min. At the highest value of blood flow rate, it can be observed that the endothelin clearance rate changes marginally. Figure 8 demonstrates the effect of blood flow rate on the clearance rate of β2-microglobulin. The β2-microglobulin clearance rate reaches the maximum value of 22 mL/min at 100 mL/min blood flow rate. The larger molecules, such as β2-microglobulin, cannot pass easily through the conventional dialysis membranes and are therefore conserved in the patient’s blood. A universal highlight of synthetic membranes is their comparatively large pore size, following by high ultrafiltration coefficients and high clearances of β2-microglobulin. These results show that the blood flow rate increases the clearance of low molecular weight solutes (urea, glucose) but does not affect the clearance of high molecular weight. The increase in clearance with the blood flow rate can be attributed to the rise of concentration difference across the membrane. The concentration gradient across the membrane drives the transport of solutes. The concentration gradient was increased by increasing the blood flow rate, which ultimately enhanced the solutes’ clearance rate.

On the other hand, the clearance of large-sized molecules was not affected much due to the higher value of steric hindrance and friction coefficient. Due to the high value of steric hindrance, the lesser volume is available for the large size molecules to pass through the cylindrical pore. The clearance ultimately achieves a maximum for each solute, independent of the flow rate, as solutes’ concentration in the boundary layer approaches gel concentration or solubility limit. This maximum clearance value is achieved faster for high molecular weight solutes.

The results exhibited a range of values comparable with the results reported in the literature. Table 5 shows that evaluation included a comparison between the clearance rate of urea and glucose of a computational tool proposed in this study and published work. The results are inconsistent with the literature data, and the differences ranges are 0.09–6.35% and 0.22–2.63% for urea clearance rate and glucose clearance rate, respectively. Table 6 shows the excellent agreement when comparing the results of the clearance rate of endothelin and β2-microglobulin from this work against published data. A comparison demonstrated the consistency with the previous studies with a maximum difference of 1.14% and 1.38% for endothelin clearance rate and β2-microglobulin clearance rate, respectively.

Analysis of variance (ANOVA) is applied to the results of urea clearance rate and glucose clearance rate extracted from the literature and proposed computational tool. The ANOVA is used to verify the statistical significance of results obtained from the computational tool. The results of the ANOVA of the urea clearance rate and glucose clearance rate are presented in Table 7 and Table 8, respectively. Results are presented as mean with 95% confidence intervals (CIs) and *p* values of 0.9472 and 0.833 of the urea and glucose clearance rates, respectively.

## 4. Conclusions

The previously existing COMSOL application, based on membrane dialysis, lacked mathematical modeling of the membrane and solutes clearance rates. Therefore, the contaminants were not defined in the previous application. In contrast to the COMSOL application, this application is based on a detailed mathematical membrane model. The stand-alone computational tool provides the clearance rate of six different toxins and module packing density. Compared with the previous application, the stand-alone computational tool of membrane dialysis enables the user to investigate the impact of membrane characteristics (i.e., tortuosity, porosity, an average diameter of pore size, number of fibers) and process parameters (i.e., blood flow rate and dialysate flow rate) on the clearance rate of different solutes. The previous application cannot be used without COMSOL Multiphysics software. Since this application was converted to a stand-alone version using a compiler, it can be used without COMSOL Multiphysics software. A CFD model tool is developed to simulate the transport processes in a hemodialyzer, providing detailed quantitative information about the clearance rates of the solutes. The combination of computer programming and the CFD simulations are used to evaluate the transport in hemodialyzers.

## Figures and Tables

**Table 1:** COMSOL versus stand-alone computational tool model parameters.

Parameter	COMSOL Application	Stand-Alone Computational Tool [24]
Diffusion Coefficient of Solute, D	$10^{-9}{\;m}^{2}/s$	$D=1.62\times 10^{-12}\;({\mathrm{MW}}^{-0.552}$)
Membrane Diffusion Coefficient, Dm	$10^{-9}{\;m}^{2}/s$	$D_{\mathrm{es},j}=\left(\frac{D_{s,i}{ɛ}_{\mathrm{mj}}}{\tau }\right)F\left(p\right)H_{D}$
The average velocity of dialysate, Uav-dia	0.5 mm/s	Determine by Continuity and Navier Stokes equation
The average velocity of permeate, Uav-per	0.8 mm/s	Determine by Continuity and Navier Stokes equation

**Table 2:** Comparison between the input parameters of applications.

Membrane Parameters	COMSOL Application	Computational Tool
The inner radius of the fiber, R1	✓	✓
Radius up to the outer layer, R2	✓	✓
The radius of the concentric permeate channel, R3	✓	✓
Length of the fiber, H	✓	✓
Tortuosity, τ	✕	✓
The porosity of the skin layer, Ɛms	✕	✓
The average diameter of the skin layer pores	✕	✓
Number of fibers, n	✕	✓
Process parameters		
Inlet concentration, $C_{0}$	✓	✓
Blood flow rate, Qb	✕	✓
Dialysate flow rate, Qd	✕	✓

**Table 3:** Results are available in a computational tool developed in this study versus the COMSOL application.

Computational Tool Application Results	COMSOL Application Results
Urea clearance rates	Contaminant concentration in dialyzed blood
Glucose clearance rate	Contaminant removal
Endothelin clearance rate	
β2-microglobulin	
Complement factor D	
Albumin	
Packing density	

**Table 4:** Default Input parameters used in computational tool.

Input Parameters	Values	Units
Membrane Parameters		
Inner radius of the fiber (R1)	0.10	mm
Radius up to outer layer (R2)	0.145	mm
Radius of concentric permeate channel (R3)	0.210	mm
Length of the fiber (H)	270	mm
Tortuosity	2.27	
Porosity of skin layer	0.1	
Average diameter of skin layer pores	39.5	mm
Number of fibers (n)	12,000	
Process Parameters		
Inlet concentration (c0)	1	mol/L
Blood flow rate (Qb)	300	mol/L
Dialysate flow rate (Qd)	500	mol/L

**Table 5:** Comparison of clearance rate of urea and glucose at different blood flow rates with literature (dialysate flow rate (Qd) = 500 mL/min).

Blood Flow Rate (mL/min)	Urea Clearance Rate [15] (mL/min)	Urea Clearance Rate [This Study] (mL/min)	Difference (%)	Glucose Clearance Rate [15] (mL/min)	Glucose Clearance Rate [This Study] (mL/min)	Difference (%)
200	187	186.2	0.43	151	152.5	0.99
250	218	220	0.92	170	169.8	0.12
300	245	247.8	1.14	183	182.6	0.22
350	269	270.6	0.59	195	192.5	1.28
400	288	289.5	0.52	203	200.2	1.38
450	305	305.2	0.07	209	206.2	1.34
500	340	318.4	6.35	215	211	1.86
550	330	329.7	0.09	219	214.9	1.87
600	340	339.3	0.21	224	218.1	2.63

**Table 6:** Comparison of clearance rate of endothelin and β2-microglobulin at different dialysate flow rates with literature (blood flow rate (Qb) = 400 mL/min).

Dialysate Flow Rate (mL/min)	Endothelin Clearance Rate [15] (mL/min)	Endothelin Clearance Rate [This Study] (mL/min)	Difference (%)	β2-Microglobulin Clearance Rate [15] (mL/min)	β2-Microglobulin Clearance Rate [This Study] (mL/min)	Difference (%)
200	42.8	38.95	9.00	24.07	20.63	14.29
300	43.55	39.51	9.28	24.32	20.89	14.10
400	43.67	39.86	8.72	24.57	21.08	14.20
500	43.8	40.14	8.36	24.32	21.22	12.75
600	43.8	40.39	7.79	24.69	21.32	13.65
700	44.04	40.59	7.83	24.81	21.4	13.74

**Table 7:** One-Way Analysis of Variance (ANOVA) of urea clearance rate.

Data Summary (Urea Clearance Rate)					
Groups		N	Mean	Std. Dev.	Std. Error
Group 1 (Urea clearance rate [15] (mL/min))		9	280.2222	55.0313	18.3438
Group 2 Urea clearance rate [This study] (mL/min))		9	278.5222	52.0702	17.3567
ANOVA Summary (urea clearance rate)					
Source	Degrees of Freedom DF	Sum of Squares SS	Mean Square MS	F-Statistics Value	*p*-Value
Between Groups	1	13.005	13.005	0.0045	0.9472
Within Groups	16	45,917.9977	2869.8749		
Total:	17	45,931.0027			

**Table 8:** One-Way Analysis of Variance (ANOVA) of glucose clearance rate.

Data Summary (Glucose Clearance Rate)					
Groups		N	Mean	Std. Dev.	Std. Error
Group 1 (Glucose clearance rate [15] (mL/min))		9	196.5556	24.3932	8.1311
Group 2 (Glucose clearance rate [This study] (mL/min))		9	194.2	22.1744	7.3915
ANOVA Summary (Urea clearance rate)					
Source	Degrees of Freedom DF	Sum of Squares SS	Mean Square MS	F-Statistics Value	*p*-Value
Between Groups	1	24.9698	24.9698	0.046	0.833
Within Groups	16	8693.8578	543.3661		
Total:	17	8718.8276			

## Data Availability

Not applicable.

## Supplementary Materials

The following are available online at https://www.mdpi.com/article/10.3390/membranes11120916/s1, Figure S1: Switching from model builder to application builder; Figure S2: Process of form development; Figure S3: Input Form developed with Form Objects—Line, Text Label, Input Field and Unit; Figure S4: Results Form developed with Form Objects—Text Label, Data Display, and Unit; Figure S5: Graphics window developed with Forms 3–6; Figure S6: Description Form developed with Form Object—Image; Figure S7: Info Form developed with Form Objects—Text Label, Line, Data Display, Information Card Stack; Figure S8: Main Form developed with Form Objects—Text Label and Form Collection; Figure S9: New Method nodes created under Methods; Figure S10: Method 1 Developed to generate an automatic report; Figure S11: Method 2 developed to check the feasibility of the solution; Figure S12: Method 3 developed to check the feasibility of the solution; Figure S13: Method 4 developed to incorporate important graphical results; Figure S14: Method 5 developed to incorporate a tabular form of the result table in report; Figure S15: Steps for development of the file menu and ribbon tab using the main window; Figure S16: Adding path of file using library files setting window; Figure S17: Compiler available in the main section of the home tab.
