# Supplementary material for: Design and Development of a Computational Tool for a Dialyzer by Using Computational Fluid Dynamic (CFD) Model
Source: Membranes (Basel). 2021 Nov 24;11(12):916. doi: 10.3390/membranes11120916 (PMC8706063; doi:10.3390/membranes11120916)
Supplement: Supplementary file 1 [file membranes-11-00916-s001.zip › membranes-1248963-supplementary.pdf]

membranes

Supplementary Materials

Design and Development of a Computational Tool for a Dialyzer by using Computational Fluid Dynamic (CFD) model

Tuba Yaqoob, Muhammad Ahsan*, Sarah Farrukh and Iftikhar Ahmad

[1] School of Chemical and Materials Engineering, National University of Sciences and Technology, Islamabad 44000, Pakistan; tyaqoob.pse1@scme.nust.edu.pk, ahsan@scme.nust.edu.pk, sarah.farrukh@scme.nust.edu.pk, iftikhar.salarzai@scme.nust.edu.pk,

* Correspondence: ahsan@scme.nust.edu.pk; Tel.: +92-51-90855125

1. Switching to COMSOL Application Builder

In order to develop the application, COMSOL Application Builder was selected, as shown in Figure 1. The main entities under the Application builder window used to build this application are Main Window, Forms, Methods, and Libraries.

1.1. Application Development

Figure 1 represents the scheme of workflow followed to build the application. Stepwise development of application is as follows:

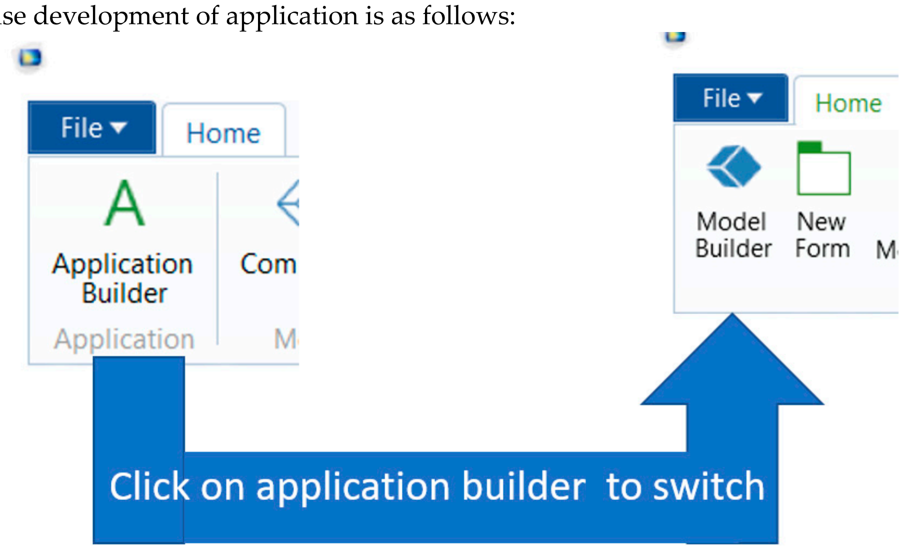

Figure S1. Switching from model builder to application builder

1.2. Development of Form

The application user interphase was developed by creating nine different Forms. As soon as the new form is created, Form Objects become available on the ribbon tab and inserted various entities on the Forms. The form objects window is shown in Figure 2. It is essential to mention that each **Form Object** has a setting window that opens on inserting the object. The development of the forms is illustrated below.

1.2.1. Form 1-Input

Form 1 was created to develop a list of all the input parameters available to the user. A grid layout containing three columns and thirteen rows was created (Figure 3). In the first and tenth row, Line was inserted from Form Objects to put Membrane Parameters and Process Parameters' headings. In order to write the names of parameters, the remaining rows of the first column were inserted with Text Label. The middle column was added with Input Field to create a placeholder for the user, where the user can put a desired

value of the parameter. Each row of the third column was inserted with the **Unit** to put the units of input parameters.

1.2.2. Form 2-Results

It can be seen in Figure 4; Form 2 was dedicated to showing the impact of the changes made in the input parameters on the clearance rate of various solutes. In the form's setting window, the grid layout was created with three columns and seven rows. Each row of the first, second, and third columns was inserted with Text Label, Data Display, and Unit. Text Label was used to enter the names of output parameters in the first column. In contrast, Data Display was used to show the output parameter's numeric value in the second column. The units of output parameters were inserted in the last column with the help of the Unit.

1.2.3. Form 3-geodraw

As shown in Figure 5, Form 3 was created to create a graphics window on the user interphase to show basic plots. In this form's setting window, only one cell was created, and Graphics was inserted in this cell. In the Selected source dialogue box, Geometry Display was added from the Source for Initial Graphic Content.

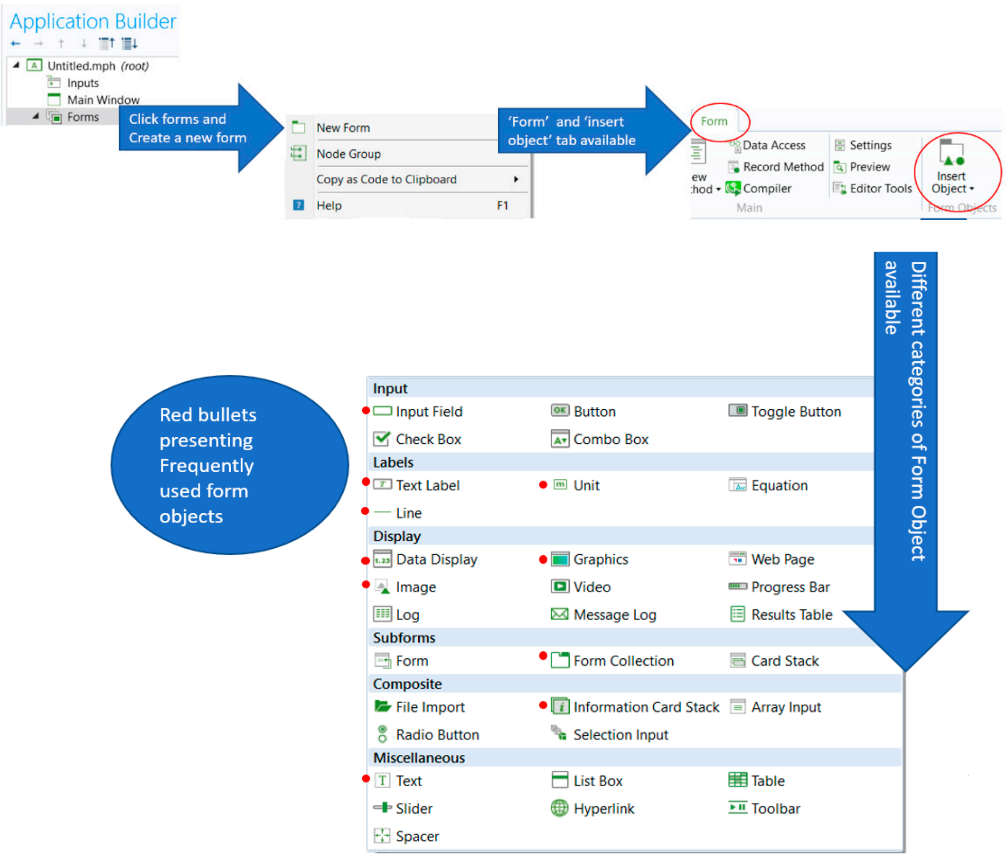

Figure S2. Process of Form development.

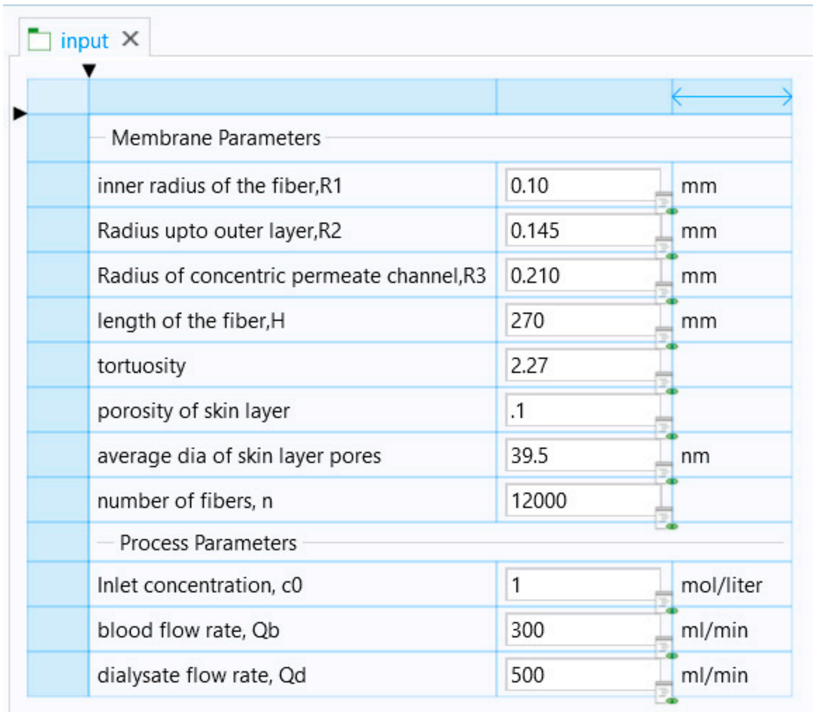

input

**Membrane Parameters**		
inner radius of the fiber,R1	0.10	mm
Radius upto outer layer,R2	0.145	mm
Radius of concentric permeate channel,R3	0.210	mm
length of the fiber,H	270	mm
tortuosity	2.27	
porosity of skin layer	.1	
average dia of skin layer pores	39.5	nm
number of fibers, n	12000	
**Process Parameters**		
Inlet concentration, c0	1	mol/liter
blood flow rate, Qb	300	ml/min
dialysate flow rate, Qd	500	ml/min

Figure S3. Input Form developed with Form Objects-Line, Text Label, Input Field and Unit.

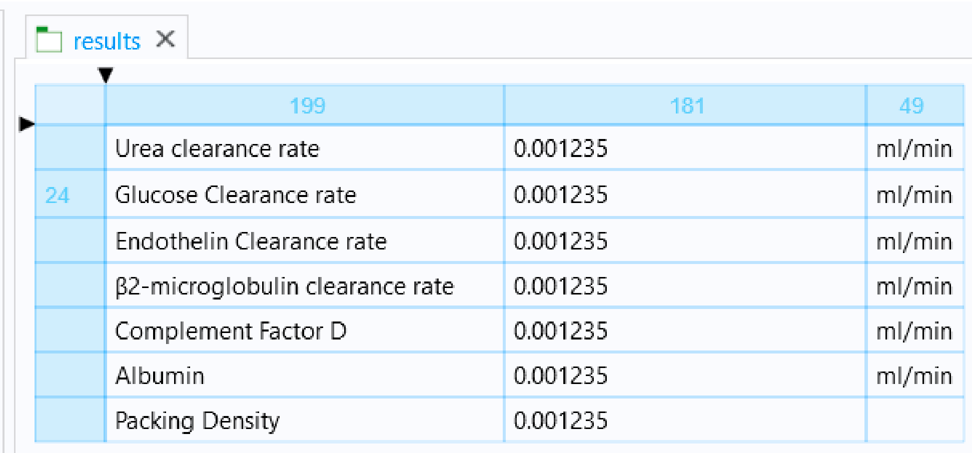

results

	199	181	49
	Urea clearance rate	0.001235	ml/min
24	Glucose Clearance rate	0.001235	ml/min
	Endothelin Clearance rate	0.001235	ml/min
	β2-microglobulin clearance rate	0.001235	ml/min
	Complement Factor D	0.001235	ml/min
	Albumin	0.001235	ml/min
	Packing Density	0.001235	

Figure S4. Results Form developed with Form Objects-Text Label, Data Display, and Unit.

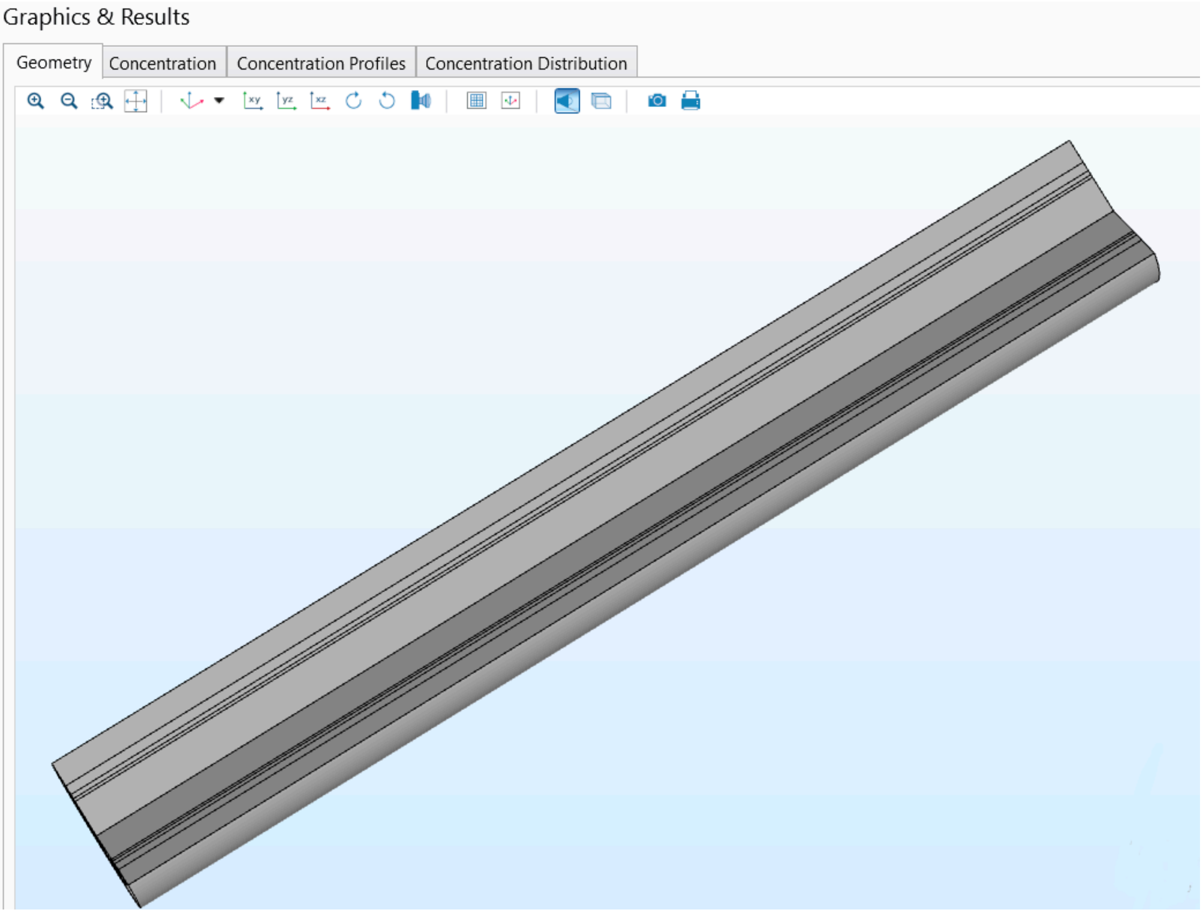

Figure S5. Graphics window developed with Form 3-6.

1.2.4. Form 4,5,6-concentration, concentration profile, concentration distribution

These forms were created to display three different plots in the graphics window, including concentration, concentration profile, and concentration distribution, as shown in Figure 5. The settings and Form Object of these forms were identical to Form 3.

1.2.5. Form 7- Description

The form was created to add an image of a two-dimensional axisymmetric model of the membrane, as shown in Figure 6. In order to insert the Image, one cell was created, and Image was inserted in the cell from Form Object. Since the model image was not available in the default list of images, the Image was first added and then inserted from the Image's dropdown menu.

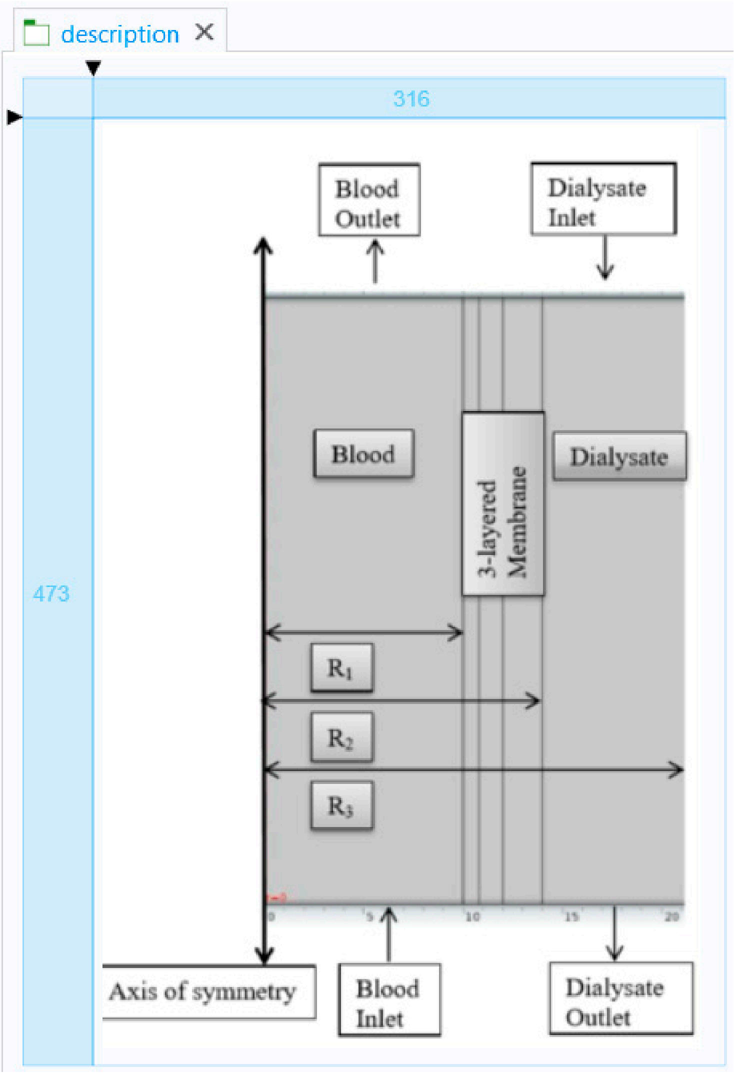

Figure S6. Description Form developed with Form Object -Image.

1.2.6. Form 8- Info

As shown in Figure S7, it was created with two columns and three rows to add expected and last computation time. Text Label and Line were used to insert text, i.e., Expected computation time and Status, respectively. Data Display and Information Card Stack were used to display the numeric values of expected computation time and last computation time.

1.2.7. Form 9- Main

It is evident from Figure 8 that this form was created to develop the user interphase of the application. For this purpose, all the forms mentioned above were inserted in different cells of this form. The grid layout of this form comprised four rows and four columns, in which the last three rows of the first column were merged. In the first and third column of the second row, Form Collection was inserted. In Form Collection of column 1, Form 1- Input, Form 7- Description and Form 2- Results were kept in the selected pane for display. Similarly, in the Form Collection of column 3, Form 3-geodraw and Form 4,5,6-concentration, concentration profile, concentration distribution were kept in the selected pane for display. In the first, third, and fourth columns of the top row, headings, i.e., Input & Results and Graphics & Results, and the institute's logo were added, respectively.

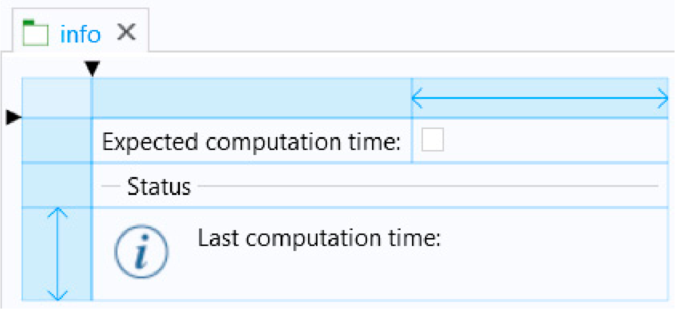

Figure S7. Info Form developed with Form Objects-Text Label, Line, Data Display, Information Card Stack.

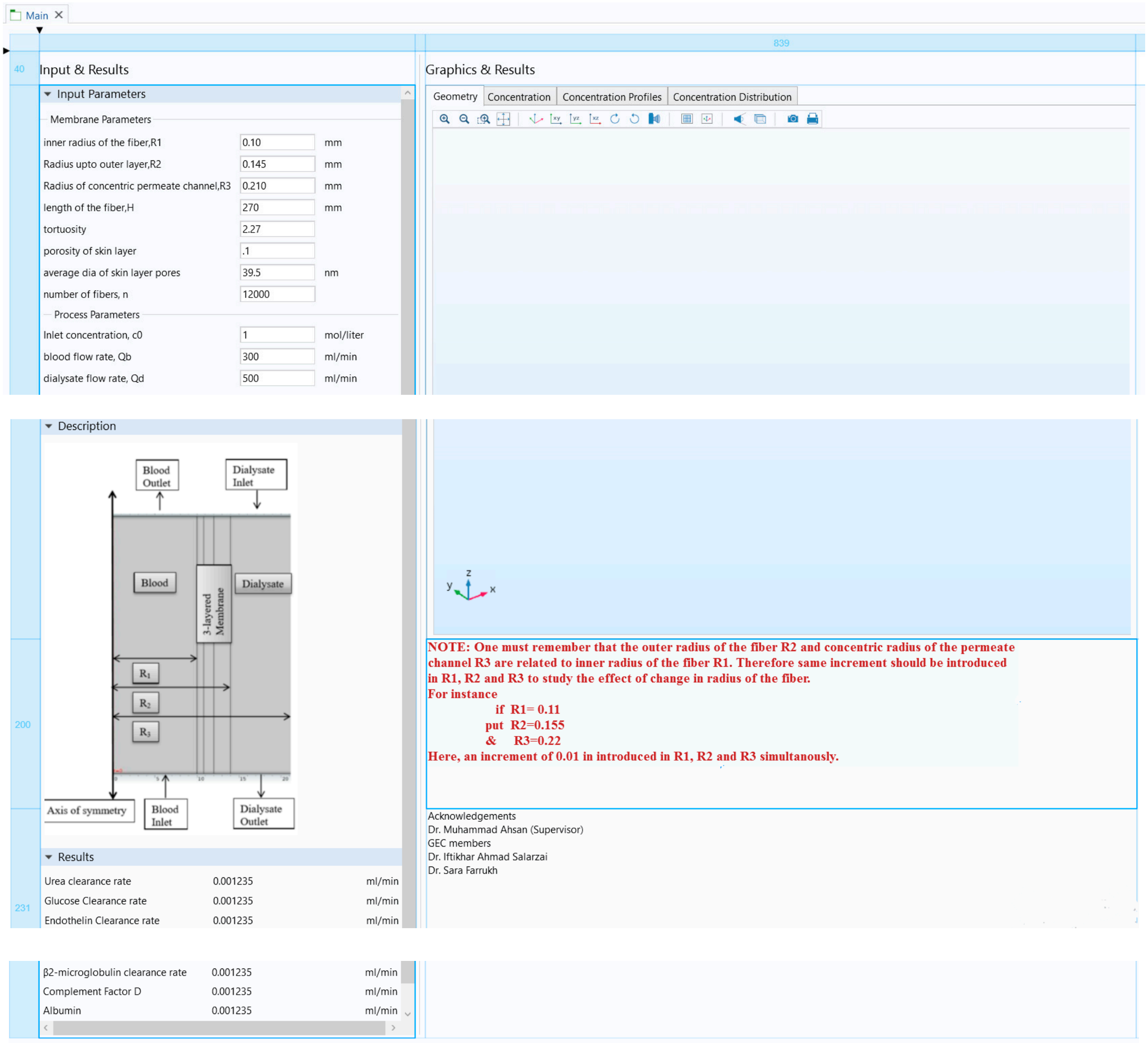

Figure S8. Main Form developed with Form Objects-Text Label and Form Collection.

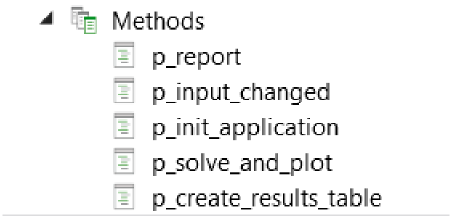

Figure S9. New Method nodes created under Methods.

1.3. Development of Methods

As shown in Figure 9, five new methods were created under the Application Builder window's Method node to perform actions that were not included in the default computational commands. From the Language Elements available on the ribbon, block statements and array operations were used to make loops and actions. The following methods were developed to build this application.

1.3.1. Method 1-p report

This method was created to include a report of the results in the application. The code written in *Method 1* is shown in Figure 10. If-else block statement was used to alert the user that the user must press compute before generating the report if the input is changed. The commands for the report generation were given in the Model Builder environment. Under the Report node, one Title Page and three Sections were created in the Model Builder window. The front matter of the Report was set up in the setting window of the Title Page. A summary of membrane dialysis was also included through the Title Page settings window. Under each Section node, other Section were included to add Model content, i.e., root, parameters, table of the result showing blood concentration and concentration profiles, in the report.

1.3.2. Method 2- p input changed

The code written in this Method node is shown in Figure 11. This code confirms the feasibility of the solution on the input parameters; so that the application computes only correct numeric inputs.

1.3.3. Method 3- p init application

The code of this Method node is shown in Figure 12. It was written to automatically change the results of the graphics window according to the simulated input values.

1.3.4. Method 4- p solve and plot

It can be seen in Figure 13, and this Method node was created to write a code for including desired plots in the graphics window.

1.3.5. Method 5- p create a result table

In order to include the tabular form of the results in the report, the code written is shown in Figure 14. In this method, the clearance rate of toxins and the dialyzer's packing density are included in the report.

1.4. Main Window

The primary Window node was used to include a Ribbon Tab and File Menu in the application. It can be seen in Figure 15 that these entities are available on the ribbon of the application building environment. Under the File Menu, two Items were included to create the options of save and save as. Under the Ribbon tab, one Item was added to create a Home tab. Under the home (Item), four Ribbon Section were included to develop Input, Geometry, Simulation, and Documentation sections in the application. The options created under these sections are Reset to Default, Update, Compute, Report and Open PDF Documentation.

1.5. Adding the file to Libraries

In order to make a PDF document available (to provide the knowledge about the membrane model) for the user guide, a file was included under the **Library** node. In this node, the document's path is added through the file settings window, as shown in Figure 16.

1.6. Conversion to a stand-alone computational tool

In order to convert the COMSOL application to a stand-alone computational tool, the **Compiler** was added from the ribbon of the application building environment. An application that could be executable without COMSOL Multiphysics software was created through the compiler's settings window. Figure 17 shows the settings of the compiler settings window.

p_report

```
if (!solution_state.equals("solutionexists")) {
  alert("New input data. Compute to update results first.");
}
else {
  model.result().report("rpt1").run();
}
```

Figure S10. Method 1 Developed to generate an automatic report.

p_input_changed

```
if (solution_state.equals("solutionexists")) {
  solution_state = "inputchanged";
}
```

Figure S11. Method 2 developed to check the feasibility of the solution.

p_init_application

```
if (model.sol("sol1").isEmpty()) {
  solution_state = "nosolution";
}
else {
  solution_state = "solutionexists";
}

zoomExtents("geomdraw/graphics1");
zoomExtents("concentration/graphics1");
```

Figure S12. Method 3 developed to check the feasibility of the solution.

p_solve_and_plot

```
model.study("std1").run();
solution_state = "solutionexists";

useGraphics(model.result("pg116"), "concentration/graphics1");
useGraphics(model.result("pg122"), "concentration_profile/graphics1");
useGraphics(model.result("pg11"), "concentration_distribution/graphics1");
```

Figure S13. Method 4 developed to incorporate important graphical results.

p_create_results_table

```
model.result().table("tbl1").clearTableData();
model.result().numerical("min1").set("table", "tbl1");
model.result().numerical("min1").setResult();
model.result().numerical("min2").set("table", "tbl1");
model.result().numerical("min2").setResult();
model.result().numerical("min3").set("table", "tbl1");
model.result().numerical("min3").setResult();
model.result().numerical("min4").set("table", "tbl1");
model.result().numerical("min4").setResult();
model.result().numerical("min5").set("table", "tbl1");
model.result().numerical("min5").setResult();
model.result().numerical("min6").set("table", "tbl1");
model.result().numerical("min6").setResult();
model.result().numerical("min7").set("table", "tbl1");
model.result().numerical("min7").appendResult();
```

Figure S14. Method 5 developed to incorporate a tabular form of the result table in report

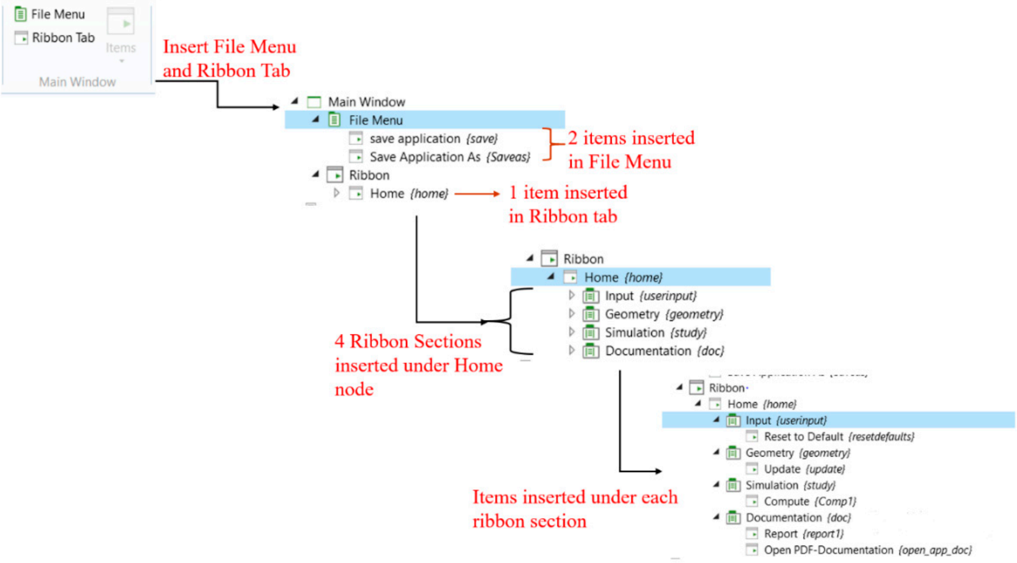

.

Figure S15. Steps for development of the file menu and ribbon tab using the main window.

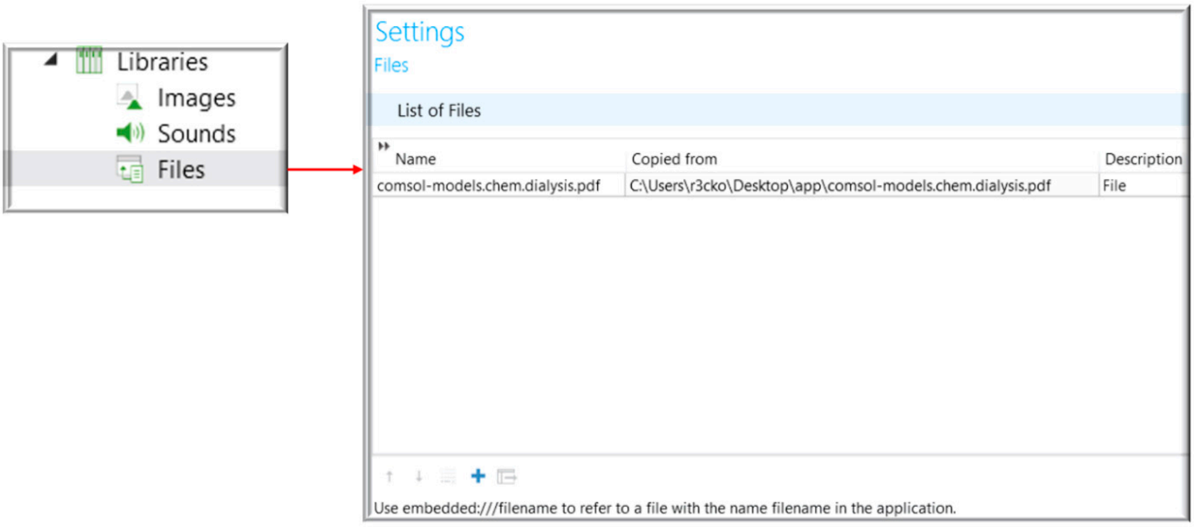

Figure S16. Adding path of file using Library files setting window.

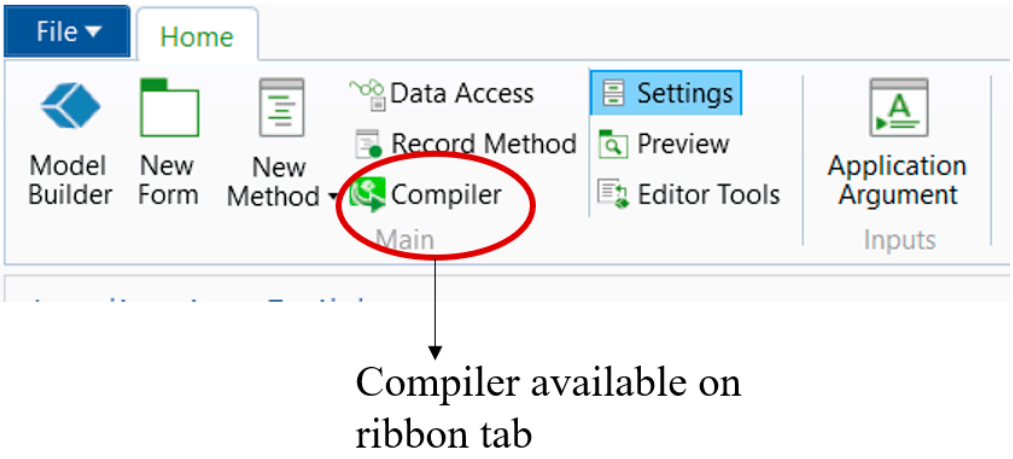

Figure S17. Compiler available in the main Section of the Home tab.
